# Chytrid Fungus in Europe

**DOI:** 10.3201/eid1110.050109

**Published:** 2005-10

**Authors:** Trenton W.J. Garner, Susan Walker, Jaime Bosch, Alex D. Hyatt, Andrew A. Cunningham, Matthew C. Fisher

**Affiliations:** * Zoological Society of London, London, United Kingdom; † Imperial College, London, United Kingdom; ‡ Museo Nacional de Ciencias Naturales, Madrid, Spain; § Commonwealth Scientific and Industrial Research Organisation, Geelong, Victoria, Australia

**Keywords:** molecular epidemiology, infectious diseases, emerging, letter

**To the Editor:** Amphibian species are declining at an alarming rate on a global scale (*1*). One of the major reasons for these declines is chytridiomycosis, caused by the chytridiomycete fungus, *Batrachochytrium dendrobatidis* (*1**,**2*). This pathogen of amphibians has recently emerged globally (*2**,**3*) and has caused mass die-offs and extensive species declines on 4 continents (*1**,**3*); knowledge of its distribution and effects on amphibian populations remains poor. In Europe, little is known about *B. dendrobatidis* distribution, which is disturbing when one considers that at least 3 European amphibian species are undergoing chytrid-associated die-offs that will likely lead to local extinction (*4**,**5*) (J. Bosch et al., unpub. data).

We screened 1,664 current and archived samples of wild amphibians collected in Europe from 1994 to 2004 by researchers using amphibians as study organisms. *B. dendrobatidis* infects the skin of adult amphibians and the mouthparts of anuran larvae; samples included toe clippings and skin samples from adults and mouthparts of tadpoles. Our sampling was opportunistic, including both caudates and anurans. We screened all samples for chytrid fungus with quantitative real-time polymerase chain reaction (PCR) of the ITS-1/5.8S ribosomal DNA region of *B. dendrobatidis* (*6*), including appropriate positive and negative controls. We confirmed real-time PCR positives by amplifying a subset of these positives with a second *B. dendrobatidis*–specific PCR with a nested reaction developed from the *ctsyn1* locus (*3*). To confirm that detection with real-time PCR indicated a viable chytrid infection, when actual tissue samples were available, we examined a generous subset using histologic features for typical signals of pathogenic *B. dendrobatidis* infection. Specifically, we found intracellular zoospore-carrying sporangia within the stratum corneum and stratum granulosum of toe and skin samples. We also compared real-time PCR amplification profiles of suspected positives to those generated from samples from animals involved in chytrid-driven die-offs and found these results to be comparable. Furthermore, attempts to isolate the fungus from dead animals were successful when animals were obtained in a suitable condition for this purpose (see below).

Our survey found *B. dendrobatidis* in amphibians in 5 European countries, Spain, Portugal, Italy, Switzerland, and Great Britain. Previously, chytrid infection has been reported in wild amphibians only in Spain, Germany, and Italy (*4**,**5**,**7**,**8*). We detected chytrid fungus in 20 of 28 amphibian species examined, representing 9 different genera, 5 anuran, and 4 caudate, in 6 families. We found signs of chytrid in archived samples from as early as 1998. The number of infections per country we found were Austria 0/24, Croatia 0/8, Czech Republic 0/18, Italy 2/101, France 0/60, Germany 0/51, Greece 0/88, Portugal 1/25, Slovenia 0/29, Spain 108/345, Sweden 0/197, Switzerland 63/252, and United Kingdom 2/466. Infection prevalence was exceptionally high in Spain and Switzerland. In Spain, ongoing chytridiomycosis-driven declines of midwife toads (*Alytes obstetricans*) and salamanders (*Salamandra salamandra*) have been documented since 1997 (*4*) and 1999 (*5*), respectively, and confirmed with scanning electron microscopy, histologic examination, and molecular detection methods (*4**,**5*). Common toads have been suffering apparently minor chytrid-related die-offs in Spain for several years, but mass die-offs were observed in 2004 (*5*) (J. Bosch et al., unpub. data). No chytrid-related die-offs have been reported in Switzerland. Furthermore, the infected animals from Switzerland were all adults in good breeding condition, many of which reproduced successfully in behavioral and ecologic experiments. Real-time PCR amplification profiles for the Swiss samples were quantitatively equivalent to those generated from samples of *A. obstetricans* collected during mass die-off events in Spain; from these latter samples, we successfully isolated viable *B. dendrobatidis* cultures from 2 geographically distinct areas. In Great Britain, we found chytrid in 2 of 14 introduced North American bullfrogs (*Rana catesbeiana*) caught in 2004 but did not find it in wild-captured native species. Examination by microscope and electron microscope of 180 native British amphibians from 1992 to 1996 did not find chytrid infection (A.A. Cunningham, unpub.data). The ability of the North American bullfrog to act as a vector for chytrid range expansion has been hypothesized (*9**,**10*). Our data may indicate that bullfrogs can fulfill this role in Great Britain and other areas; we have found the molecular signal of chytrid infection from introduced North American bullfrogs collected on 3 separate continents (T.W.J. Garner et al., unpub. data).

This survey shows that *B. dendrobatidis* is widely and irregularly distributed in Europe and infects a broad range of amphibian species. Furthermore, because of the opportunistic nature of our sampling strategy, our results certainly underestimate the overall prevalence of *B. dendrobatidis* in Europe. These findings are surprising considering that chytrid-related die-offs have been infrequently described in Europe. This may be because *B. dendrobatidis* has only recently and rapidly expanded its range into Europe (*3*), and the consequences are only now being detected in wild amphibian populations; because the expression of chytridiomycosis is environmentally limited (*11*); or because European amphibians exhibit highly variable levels of resistance to chytrid infection. Notwithstanding, our knowledge of the epidemiology of *B. dendrobatidis* is insufficient to effectively manage wildlife and conduct disease abatement. As data regarding the distribution of chytrid fungus accumulate and the ecologic requirements for disease persistence and transmission are identified (*11*), management of the pathogen can become more predictive. Basic management practices, such as restricting transportation of potential carriers and restricting pet trading and reintroduction projects, coupled with field monitoring, must be improved to prevent further global emergence of this pathogen. Our results also show that asymptomatic amphibians must be included in any broad-scale epidemiologic screening for this emergent pathogen.
